# Hypoxia-inducible miR-210 contributes to preeclampsia via targeting thrombospondin type I domain containing 7A

**DOI:** 10.1038/srep19588

**Published:** 2016-01-22

**Authors:** Rongcan Luo, Yongqing Wang, Peng Xu, Guangming Cao, Yangyu Zhao, Xuan Shao, Yu-xia Li, Cheng Chang, Chun Peng, Yan-ling Wang

**Affiliations:** 1 State Key Laboratory of Reproductive Biology, Institute of Zoology, Chinese Academy of Sciences, Beijing 100101, China; 2 Department of Obstetrics and Gynaecology, Peking University Third Hospital, Beijing 100191, China; 3 Department of Biology, York University, Toronto, Ontario, Canada; 4 School of Life Sciences, Lanzhou University, Lanzhou 730000, China; 5 College of Life Sciences, Shanxi University, Taiyuan, Shanxi 030006, China; 6 University of Chinese Academy of Sciences, Beijing 100049, China

## Abstract

Preeclampsia, a relatively common pregnancy disorder, is a major contributor to maternal mortality and morbidity worldwide. An elevation in microRNA-210 (miR-210) expression in the placenta has been reported to be associated with preeclampsia. Our bioinformatic analysis showed that thrombospondin type I domain containing 7A (THSD7A) is a predicted target for miR-210. The aim of this study was to determine whether miR-210 is involved in preeclampsia through its targeting of THSD7A in human placental trophoblasts. In preeclamptic placental tissues, THSD7A levels were significantly downregulated, and were inversely correlated with the levels of miR-210. THSD7A was validated as a direct target of miR-210 using quantitative real time PCR (qRT-PCR), Western blotting, and dual luciferase assays in HTR8/SVneo cells. Transwell insert invasion assays showed that THSD7A mediated the invasion-inhibitory effect of miR-210 in HTR8/SVneo cells. Interestingly, hypoxia markedly increased miR-210 expression while suppressing THSD7A expression in a time-dependent manner in HTR8/SVneo cells. This study provides novel data on the function of THSD7A in human placental cells, and extends our knowledge of how miR-210 is involved in the development of the preeclampsia.

MicroRNAs (miRNAs) are non-coding RNAs of approximately 22 nucleotides that post-transcriptionally regulate gene expression by forming imperfect base pairing with sequences in the 3′untranslated region (3′UTR) of target genes; thereby preventing protein production by inducing mRNA degradation or by repressing translation^1^,^2^. Approximately 30% of human protein-coding genes can be regulated by miRNAs^3^. MiRNAs have been found to be involved in diverse biological processes, including cell proliferation, differentiation, apoptosis, and angiogenesis^1^,^4^.

Affecting 3–5% of pregnancies, preeclampsia, a pregnancy-associated disorder, is a major contributor to maternal mortality and morbidity worldwide^5^,^6^. The pathogenesis of this disorder is not clearly understood, although it has been generally believed that abnormal development of the placenta at early gestation may be the critical cause of the disease^7^. Specifically, impaired trophoblast invasion and aberrant spiral arterial remodelling is thought to result in decreased utero-placental perfusion and excessive placental hypoxia/ischemia, causing the release of soluble factors or cell debris which further damage the maternal endothelium^8^. Recently, accumulating evidence has indicated that the abnormal expression of miRNAs in the placenta is associated with preeclampsia^9^,^10^,^11^,^12^,^13^,^14^,^15^,^16^. Interestingly, previous studies by us and others have demonstrated that miR-210 is predominantly elevated in placental tissues and maternal plasma of patients with preeclampsia^9^,^10^,^11^,^12^,^13^,^14^,^15^,^16^,^17^,^18^. Elucidation of the underlying mechanisms by which miR-210 exerts its effects in placental cells is likely to provide a better understanding of the pathophysiology of the disorder, and uncover new targets for therapeutic intervention.

We performed bioinformatic analysis to predict target genes for miR-210 using TargetScan (http://www.targetscan.org/vert_61/), and found that thrombospondin type I domain containing 7A (THSD7A) was one of the most common predicted targets. THSD7A has been shown to be expressed in placenta vasculature and in human umbilicalvein endothelial cells (HUVECs)^19^; however, its role in the placenta is unknown. The THSD7A protein has a net molecular weight of approximately 186-kDa, and approximately 260-kDa following post-translational modification by extensive N-glycosylation^20^. It contains multiple thrombospondin type I repeats (TSRs) and RGD motif, and can work as an extracellular matrix (ECM) protein to regulate the interaction of cells with other ECMs^21^. The cooperation between its TSRs and integrin α_V_β_3_ can promote endothelial cell motility and melanoma cell invasion^22^. In HUVECs and SH-SY5Y neuroblastoma cells, a 210-kDa soluble form of THSD7A protein can be released, and this soluble THSD7A promotes endothelial filopodia formation and focal adhesion assembly and induces FAK-dependent signalling during angiogenesis^20^.

Based on this evidence, we hypothesized that miR-210 may participate in the regulation of trophoblast function in part by targeting THSD7A. To test the hypothesis, the localization of THSD7A in human placenta and the differential expression of THSD7A in preeclamptic placentas were examined. The targeting and repression of THSD7A by miR-210 were validated by a luciferase reporter assay in a human placental trophoblast cell line, HTR8/SVneo. In addition, the influence of miR-210 and THSD7A on trophoblast cell invasion was further explored. We also examined the regulation of THSD7A by hypoxia. The findings reported in the present study highlight the crucial role of miR-210 in human placental trophoblasts, and provide new insight into the pathogenesis of preeclampsia.

## Results

### THSD7A was down-regulated in severe preeclamptic placenta

**Figure 1:**
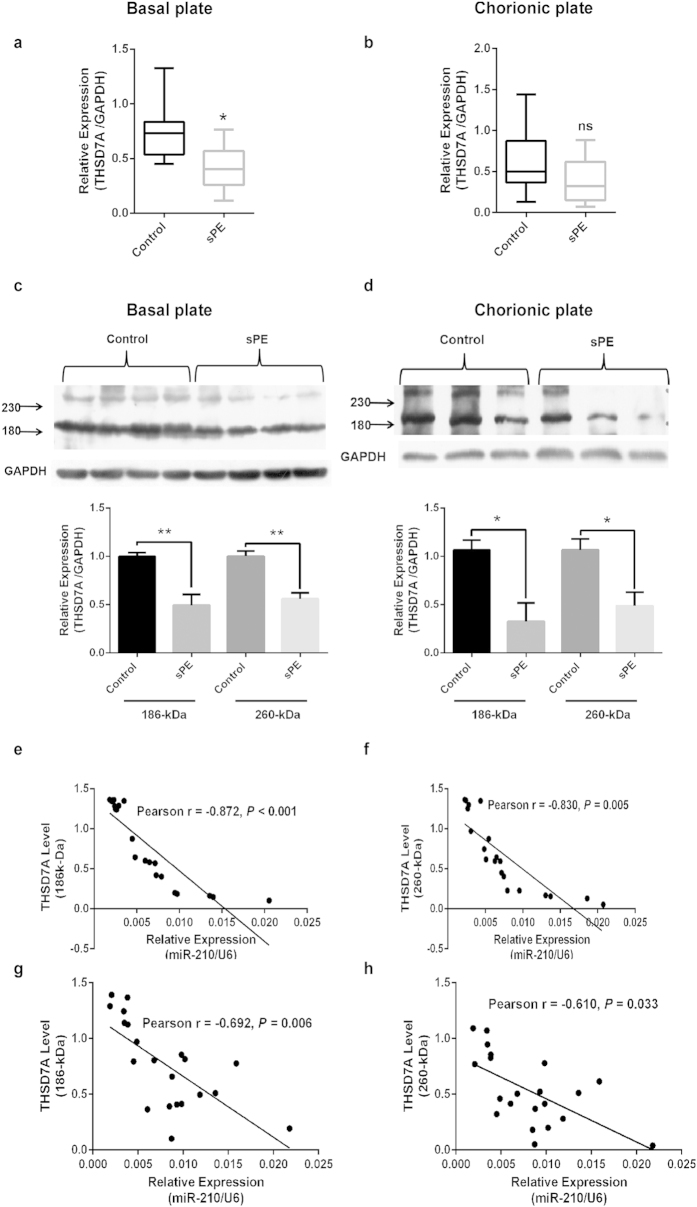
Expression patterns of THSD7A in placentae derived from severe preeclamptic (sPE) patients and normal pregnant women (Control). qRT-PCR (**a,b**) and Western blotting experiments (**c,d**) were performed to measure the expression level of THSD7A in the basal plate (**a,c**) and in the chorionic plate (**b,d**) of the placentae derived from sPE patients (n = 22) and from normal pregnant women (n = 13). The inverse correlation between the miR-210 and THSD7A expression (both 186-KD and 260-KD) in the placental basal (**e,g**) and chorionic (**f,h**) plates of the studied individuals is shown. The data are presented as the mean ± SEM; *indicates *P* < 0.05, and **indicates *P* < 0.01.

We compared the expression level of THSD7A in placentae from 13 severe preeclamptic patients and 22 normal pregnant women. qRT-PCR data revealed lower mRNA expressions of THSD7A in both basal and chorionic plates of severe preeclamptic placenta than that in normal pregnant controls (Fig. 1a,b). Results of Western blotting were consistent with the qRT-PCR data. Western blot analysis using anti-THSD7A detected two species, with molecular weight approximately 186-KD and 260-KD, in both the basal plate and chorionic plate. Interestingly, their levels were significantly lower in severe preeclamptic placentae when compared with the corresponding controls (Fig. 1c,d). We previously measured miR-210 expression in these specimens^17^,^23^, and a correlation analysis of miR-210 concentrations and THSD7A protein levels was performed accordingly. The data revealed an inverse correlation between miR-210 and THSD7A expression (both186-KD and 260-KD) in the placental basal and chorionic plates of the studied individuals (Fig. 1e–h).

### Localization of THSD7A in human placenta

**Figure 2:**
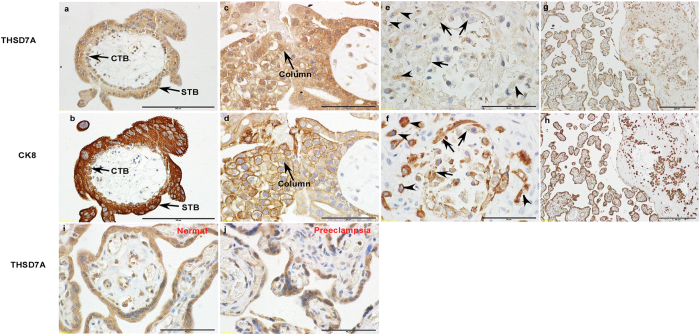
Cellular localization of THSD7A in human placenta. Immunohistochemistry was performed on paraffin sections of normal human placenta villi (**a–d**) and decidue (**e,f**) at early gestation, as well as normal (**g–i**) and preeclamptic placenta (**j**) at late gestation. Staining for cytokeratin (CK8) in the adjacent section for THSD7A was included as a trophoblast cell marker. In panel e and f, arrows indicate endovascular trophoblast cells, and arrowheads indicate interstitial extravillous trophoblast cells. CTB, cytotrophoblasts; STB, syncytiotrophoblasts; Column, column trophoblasts. Scale bars, 200 μm (**g,h**), 100 μm (**a,b**) or 50 μm (**c–f,i,j**).

Human placenta is a tissue of high heterogeneity and is composed of multiple cell types, so we performed immunohistochemistry for THSD7A in human placental tissues. At early pregnancy, the immunoreactivity for THSD7A could be predominantly observed in various subtypes of trophoblast cells, including villous cytotrophoblasts (CTB), syncytiotrophoblasts (STB), column CTBs (Column), interstitial extravillous trophoblast cells (iEVT), and endovascular extravillous trophoblast cells (enEVT) (Fig. 2a–f). The distribution pattern of THSD7A was very similar to that of miR-210, which we have previously reported^23^. The staining for THSD7A in late gestational placenta principally remained in various trophoblast cells (Fig. 2g,h), while the intensities were weaker in severe preeclamptic placenta than in normal pregnant control (Fig. 2I,j).

### Validation of THSD7A as a direct target of miR-210 in human trophoblast cells

**Figure 3:**
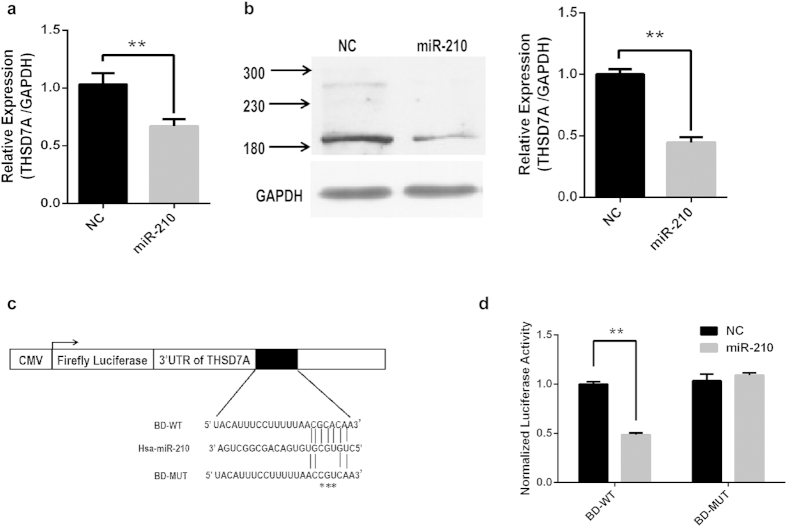
Validation of THSD7A as a direct target of miR-210 in human trophoblast cells. (**a**). THSD7A mRNA levels in HTR8/SVneo cells transfected with scramble control (NC) and miR-210 mimics (miR-210), as detected by qRT-PCR. **P < 0.01 compared with NC. (**b**). Western blot analysis to show THSD7A protein levels in HTR8/SVneo cells transfected with NC and miR-210. Left panel, a representative blot; right panel, summary graph from 3 independent experiments. The density of THSD7A was normalized to that of GAPDH in the same blot, and the values are presented as the mean ± SEM. **P < 0.01 compared with NC. (**c**). Schematic map of the luciferase reporter constructs. BD-WT contains the wild type seed sequence of miR-210 binding site in the human THSD7A 3′UTR, and BD-MUT possesses several mutated nucleotides in the seed sequence (denoted by asterisks). (**d**). Luciferase assay in HTR8/SVneo cells transfected with the BD-WT or BD-MUT reporter constructs together with miR-210 mimics (miR-210) or scrambled control (NC). The results are presented as the mean ± SEM (n =  3 independent experiments). **P < 0.01, compared with corresponding NC.

Based on our previous report^23^ and the findings presented here, miR-210 and THSD7A have a similar localization in trophoblast cells, and they exhibit an inversely related expression pattern in preeclamptic placenta. Since THSD7A is one of the predicted miR-210 target genes, we validated the targeting of THSD7A by miR-210 in a human trophoblast cell line, HTR8/SVneo. As shown in Fig 3a,b, transfection of miR-210 mimics in HTR8/SVneo cells resulted in significant down-regulation of THSD7A expression at both the mRNA and protein levels, which decreased by approximately 40% and 60%, respectively, compared to the corresponding controls that were transfected with scrambled small RNA. To address whether or not THSD7A is a direct target of miR-210, we generated luciferase reporter constructs containing the wild type (BD-WT) or mutated (BD-MUT) THSD7A 3′UTR (Fig. 3c). HTR8/SVneo cells were transfected with BD-WT or BD-MUT reporter construct, renilla luciferase vector (pRL-TK), together with miR-210 mimics or scrambled control. Relative luciferase activity was monitored 48 hours after transfection. As shown in Fig. 3d, miR-210 mimics reduced the relative luciferase activity of the BD-WT construct by about 55% compared with scrambled control (NC). However, when the predicted miR-210 binding site was mutated, the miR-210 mimic did not affect the luciferase activity. The data validated THSD7A as a target gene of miR-210 in human trophoblast cells, and indicated that the 5182-5189nt region of 3′UTR in THSD7A gene is a binding site for miR-210.

### THSD7A mediates the invasion-inhibitory effect of miR-210 in HTR8/SVneo cells

**Figure 4:**
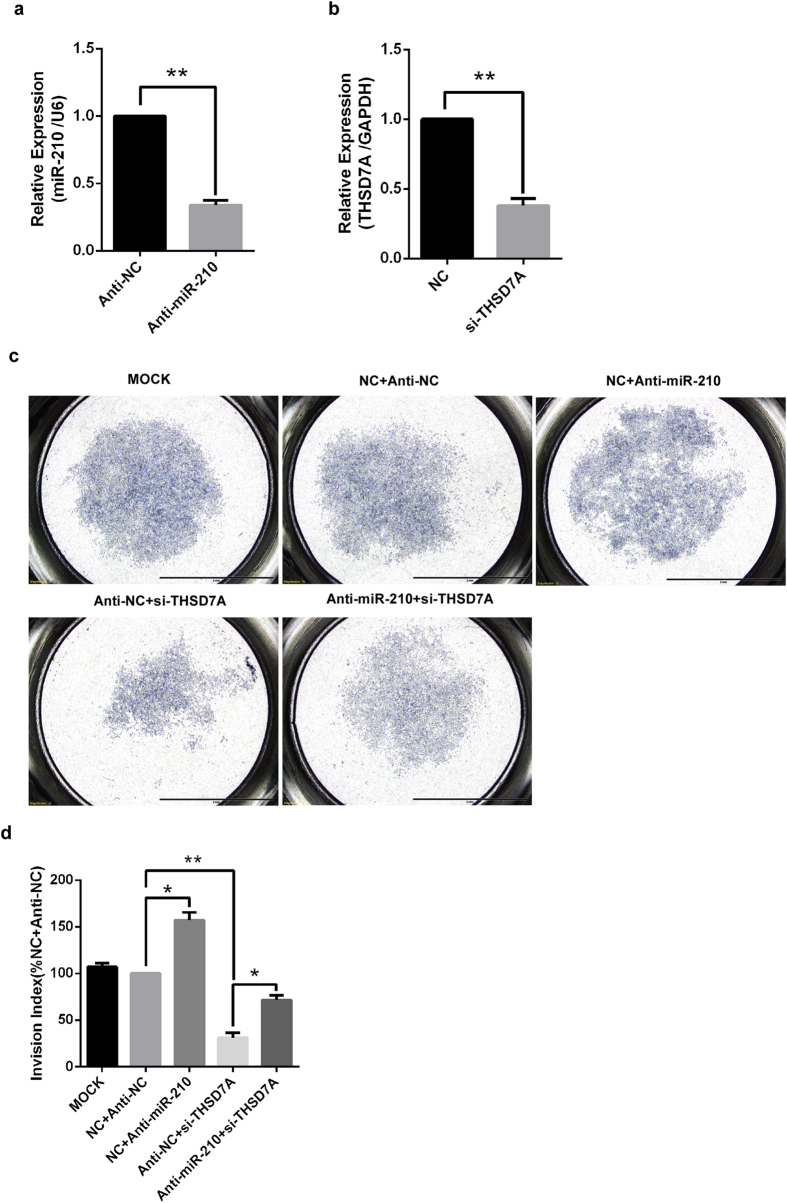
THSD7A mediates the invasion-inhibitory effect of miR-210 in HTR8/SVneo cells. (a). Anti-miR-210 decreased miR-210 levels. HTR8/SVneo cells were transfected with anti-miR-210 or its non-targeting control (anti-NC), and miR-210 levels were measured at 48 hours after transfection by qRT-PCR. The level of miR-210 was adjusted by the value of U6, and the relative value was presented as the mean ± SEM based on three independent experiments. **P < 0.01 vs NC. (**b**). Confirmation of siRNA-mediated knockdown of THSD7A. HTR8/SVneo cells were transfected with THSD7A specific siRNA (si-THSD7A) or its scramble control (NC). The level of THSD7A was determined by qRT-PCR and normalized to GAPDH. Data represent mean ± SEM of three independent experiments. **P < 0.01 vs NC. c, d. Typical result (**c**) and statistical result (**d**) of transwell invasion assays. HTR8/SVneo cells were transfected with miR-210 mimics, NC, anti-miR-210 and /or specific siRNA for THSD7A. The invasion indices were presented as the mean ± SEM based on three independent experiments. * and ** compared with the corresponding control, *P* < 0.05 and *P* < 0.01, respectively.

Consistent with previous reports that miR-210 inhibited trophoblast invasion^17^,^24^, repression of miR-210 expression with specific anti-miR promoted cell invasiveness in HTR8/SVneo cells (Fig. 4a,c). On the other hand, silencing of THSD7A expression by specific siRNAs resulted in an inhibition of cell invasion (Fig. 4b,c) and abolished the invasion-promoting effect of anti-miR-210 (Fig. 4c,d). The data demonstrate that the invasion-inhibitory effect of miR-210 in human trophoblast cells is mediated, at least in part, by THSD7A.

### Hypoxia increases miR-210 expression but down-regulates THSD7A

**Figure 5:**
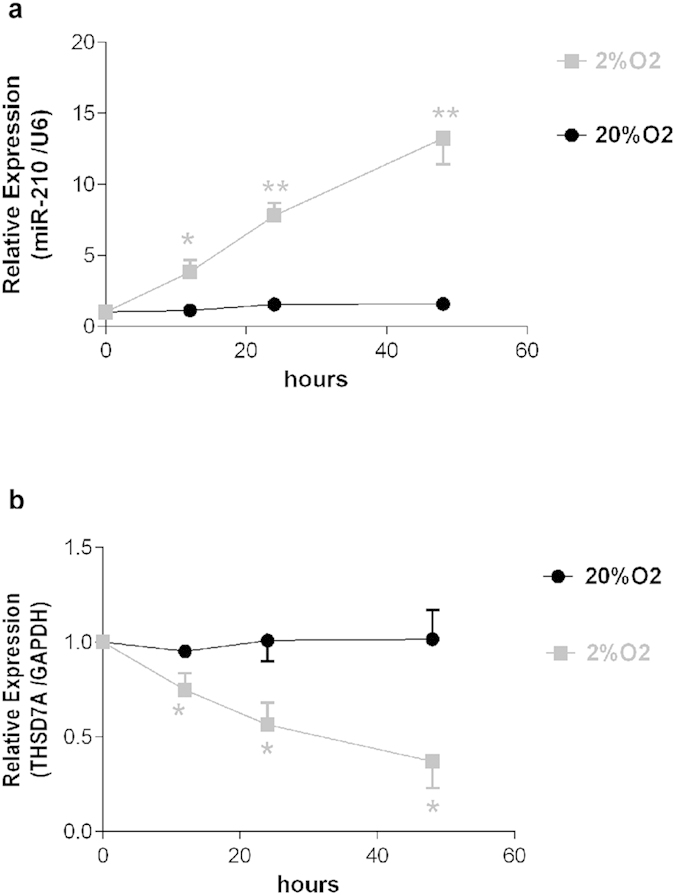
Regulation of miR-210 and THSD7A by hypoxia in HTR8/SVneo cells. HTR8/SVneo cells were cultured under 20% or 2% oxygen tension. The level of miR-210 (**a**) and THSD7A (**b**) was determined by qRT-PCR and normalized to U6 and GAPDH, respectively. Data were presented as the mean ± SEM (n = 3 independent experiments). *P < 0.05 and **P < 0.01 compared with the corresponding control.

The induction of miR-210 by hypoxia has previously been demonstrated in various cell types^18^,^25^,^26^,^27^. We kept the HTR8/SVneo cells under conditions of either normoxia (20% oxygen) or hypoxia (2% oxygen) for 48 hours, and measured miR-210 as well as THSD7A expression using qRT-PCR at various time points. As shown in Fig. 5, we observed a time-dependent, and statistically significant induction of hsa-miR-210 and a simultaneous repression of THSD7A expression by the treatment of 2% oxygen.

## Discussion

Differential expression of miRNAs between normal and preeclamptic placentae has been reported by several groups^12^,^15^,^16^,^23^, and the molecular mechanisms by which these small RNAs participate in the regulation of placental cell function has elucidated the pathogenesis of preeclampsia. MiR-210 is among the top members of a list of small RNAs with predominant overexpression in preeclamptic placentae^15^,^16^,^17^,^23^ and maternal plasma at early to mid gestation^23^. Considering the primary expression of miR-210 in placental trophoblasts, it is likely that miR-210 modulates trophoblast cell behaviour. Indeed, accumulating evidence suggest that miR-210 represses trophoblast invasion^17^,^24^, and potassium channel modulatory factor 1 (KCMF1) is one of its targets in this respect^17^. The aberrant overexpression of this small RNA may therefore contribute to the shallow trophoblast invasion and impaired spiral artery remodelling observed in preeclampsia.

Here we have validated a novel miR-210 target, THSD7A, which was also shown to be involved in modulating human trophoblast cell invasion. Several lines of evidence were presented. First, the localization of THSD7A in human placenta was similar to that of miR-210^23^. Second, the expression of THSD7A was suppressed by miR-210 in HTR8/SVneo cells. Third, the luciferase reporter assay demonstrated the direct binding of miR-210 to the 3′-UTR of THSD7A gene, with the binding site ranging from 5182nt to 5189nt. Fourth, the invasion-promoting effect of anti-miR-210 in HTR8/SVneo cells was attenuated when THSD7A expression was inhibited by siRNA. Together, these findings support the hypothesis that THSD7A is one of the functional targets of miR-210 in placental trophoblast cells. Furthermore, the expression of THSD7A in preeclamptic placentae was markedly downregulated, which was contrary to the changing expression pattern of miR-210. Upon incubation in low oxygen concentrations, miR-210 and THSD7A expression exhibited inverse responses. These observations support the data showing the correlation between the two molecules in human trophoblasts.

One of the most complex properties of microRNAs is the large number of targets that may exist for one miRNA. Based on the data from miRWalk^28^,^29^, a long list of genes are validated as targets for miR-210, including the receptor tyrosine kinase ligand ephrinA3^27^, E2F transcription factor 3^30^, the iron-sulfur cluster assembly proteins 1/2^31^, the DNA repair protein RAD52^32^, ACVR1B^33^, iron-sulfur cluster scaffold homologue (ISCU)^10^,^34^, KCMF1^17^, Ephrin-A3 and Homeobox-A9^18^, and so on. These targets mediate the effects of miR-210 in participating in oxidative stress and mitochondrial metabolism, angiogenesis^31^, cell survival^35^, DNA repair^36^, and cell migration and invasion^18^. Several target genes of miR-210 are known to be involved in preeclampsia, such as ACVR1B^16^, and studies have revealed that miR-210 could repress trophoblast cell invasion via modulating KCMF1^17^, Ephrin-A3, Homeobox-A9^18^, and THSD7A as shown in this study. We used GeneMANIA^37^ to determine how the many targeted genes might interact, and obtained a complex roadmap (Supplementary Figure 1). Although it seems that the targets may communicate in a network profile, the specific cellular microenvironment may direct the way they work. Therefore, further experimental studies are necessary to elucidate the way that miR-210 and its targets are involved in causing the placental dysfunction associated with preeclampsia.

Recently, several meta-analyses for the gene network of preeclampsia have been performed, primarily based on the comparison of genome-wide transcriptional profiling in preeclamptic and non-pathologic placentae^38^,^39^,^40^,^41^. Hypoxia, along with the oxidative stress response, appeared to be among the main affected pathways in preeclamptic placenta, and a large number of preeclampsia-associated genes, including the antiangiogenic factor sFLT-1 and endoglin, were transcriptionally regulated by the HIF-1 complex, indicating the central role played by hypoxia in the development of preeclampsia^39^,^41^. It has been well documented that oxygen partial pressure in the intervillous space varies with the development of maternal–placental circulation during pregnancy^42^,^43^. The mean oxygen concentrations at 8–10 weeks of gestation are estimated to be as low as 2–5%, and the level subsequently increases to up to 10% as the result of endovascular invasion by trophoblasts^44^. The gestational-stage-dependent variation in oxygen tension has been shown to be essential in modulating the balance of trophoblast cell proliferation and differentiation along the invasive pathway^45^,^46^. In preeclampsia, it is generally thought that the oxygen tension at the feto-maternal interface remains much lower than normal due to the shallow reconstruction of maternal blood vessels by trophoblasts. The sensitive response of miR-210 expression to hypoxia has been well-documented^36^,^47^,^48^,^49^. As miRNAs are post-transcriptional regulators that silence gene expression, placental miR-210 may be the effector of hypoxic stimulation, and may cause various changes in cell behaviour via its many target genes. In addition, the impairment of trophoblast cell invasion by miR-210 targets, including THSD7A and KCMF1, may further aggravate the hypoxic condition at the feto-maternal interface. Such an injurious feed-back loop between oxygen tension, miR-210 expression and trophoblast cell invasion may play an important role in the pathogenesis of preeclampsia.

Interestingly, studies are indicating the association between VEGF signaling and miR-210 in various cell types. In CD34^+^ endothelial cells, VEGF could dramatically increase miR-210 expression^50^, and miR-210 mimics promoted the pro-angiogenic effect of VEGF. Such a positive feedback interaction between miR-210 and VEGF signaling were observed in human synovial fibroblasts, rat osteoblast, rat kidney^51^, and a related lower plasma levels of VEGF and miR-210 was recently found in Alzheimer’s disease patients^52^. It has been well recognized that up-regulation of anti-angiogenic factors, sFlt-1 and sEndoglin, is directly involved in the occurrence of preeclampsia^53^. sflt-1 is a soluble receptor for VEGF and PlGF, which counteracts the pro-angiogenic effect of these ligands. There has been no direct link between miR-210 and sFlt-1, however, they are all sensitively up-regulated by hypoxia. One can therefore speculate that in the placenta, the oxygen tension regulated miR-210 and sFlt-1 act oppositely to keep VEGF signaling at a proper activation status. The aberrant production of miR-210 and sFlt-1 in the aspect of preeclampsia may lead to damage in angiogenesis via dysregulation in VEGF pathway.

A recent study by Vaiman and colleagues demonstrated a strong down-regulation of key hypoxia regulators including HIF-1α and HIF-1β, as well as the hypoxamir miR-210, by STOX1, a gene shown to be tightly associated with preeclampsia^54^. It is therefore likely that STOX1 overexpression mitigates an adaptive process mediated by miR-210. Mouse strains with transgenic STOX1 exhibited preeclampsia-like symptoms^55^, which substantiates the link between STOX1 and miR-210 with preeclamptic disease.

The THSD7A gene has been studied much less, and bioinformatic analysis of its links with the other miR-210 targets or the known preeclampsia-associated genes provides limited information (Supplementary Figure 2). THSD7A mRNA contains an open reading frame of 4971 nucleotides and encodes a hypothetical polypeptide of 1657 amino acids. Sequence analysis suggests the existence of an amino-terminal signal peptide followed by at least ten TSRs, one RGD motif, six tryptophan-rich sequences, one CD36-binding motif, one putative transmembrane domain, and a short cytoplasmic domain^19^. Findings from Wang *et al* revealed its preferential expression in the placental vasculature as a novel endothelial protein. They demonstrated that THSD7A could regulate cell mobility and tube formation in HUVECs, probably through the interaction of its carboxyl-terminal fragments with integrin α_V_β_3_ and paxillin^19^. Kuo *et al* demonstrated the release of a 210-kDa soluble form of the protein in HUVECs and SH-SY5Y neuroblastoma cells, which could promote endothelial filopodia formation and focal adhesion assembly, and induce FAK-dependent signalling during angiogenesis^20^. EGFR-mediated FAK signalling are key regulators of invasion and metastasis in cancer cells, and the meta-analysis studies reveal EGFR signalling as a key pathway involving the majority of differentially-regulated genes in preeclamptic placenta^41^. Therefore, it may be valuable to explore whether THSD7A functions through interaction with integrin α_V_β_3_ and FAK signalling to facilitate trophoblast cell invasion where the EGFR signalling and integrin-FAK signalling are actively involved.

In summary, the results presented extend our knowledge of the function of miR-210 and THSD7A in the human placenta, and indicate that aberrantly expressed miR-210, partly through targeting THSD7A, plays an important role in the development of the pregnancy-associated disease, preeclampsia. Further *in vivo* experiments using animal models with placenta-specific gene manipulation of miR-210 or THSD7A are warranted to comprehensively understand the role of miR-210 and its target in placenta development and their association with pregnancy outcomes.

## Materials and Methods

### Study subjects

The Ethics Committee in the Institute of Zoology, Chinese Academy of Sciences, and Peking University Third Hospital approved the study protocol for the collection of human placenta tissue samples, and written consent was obtained from all the subjects. The methods were carried out in accordance with the approved guidelines.

Tissues of human chorionic villi and decidua at gestational weeks 7–9 were obtained at Beijing Haidian Hospital (Beijing, China) from the patients who underwent therapeutic pregnancy termination. All the patients accepted no special medical treatment before termination of pregnancy. The morphological observation and pathological examination of the villi were performed to determine the gestational week of the specimens, with the record of menstrual cycles as a reference.

The placentae from normal pregnant women and severe preeclamptic patients were collected in the Department of Obstetrics and Gynaecology, Peking University Third Hospital (Beijing, China) during August 2010 to October 2012. The detailed information of the enrolled subjects was described previously^17^. A total of 22 normal pregnant subjects and 13 severe preeclamptic cases were recruited in this study. Severe preeclampsia was diagnosed according to the definition in Williams Obstetrics (23rd edition)^56^. In brief, these patients had no history of preexisting or chronic hypertension but showed systolic blood pressure of over 160 mm Hg or diastolic blood pressure over 110 mm Hg on at least two occasions, accompanied by significant proteinuria (>2 g/24 h or 3 + by dipstick in two random samples collected at >4-h interval) or problems in multiple organs (such as pulmonary oedema, seizures, oliguria, abnormal liver enzymes associated with persistent epigastric or right upper-quadrant pain, or persistent and severe CNS symptoms) after the 20th week of gestation. The clinical characteristics of the enrolled subjects are partially same as previous study^17^,^23^ and listed in Table 1. The pregnant women who developed chronic hypertension, renal disease, cardiovascular disease, gestational diabetes, spontaneous abortion, intrauterine foetal death, fetal chromosomal or congenital abnormalities or pregnancies conceived by fertility treatment were excluded from this study. All placentae were collected within 1 h of Caesarean section, and specimens at the chorionic plate and basal plate were separately taken from the placenta disc near the position of umbilical cord insertion, and were snap-frozen in liquid nitrogen. The full thickness specimens encompassing the chorionic and basal plates were subjected to routine paraffin embedding and sectioning.

### Sequences and Constructs

The siRNA duplex against human THSD7A (si-THSD7A), the mature microRNA mimics for miR-210 (miR-210), the scramble control (NC), miR-210 inhibitor (anti-miR-210) and microRNA inhibitor NC (anti-NC) were purchased from Shanghai GenePharma, China. The sequences of miRNA and siRNA are: NC, 5′-UUCUCCGAACGUGUCACGUTT-3′ (sense), and 5′-ACGUGACACGUUCGGAGAATT-3′ (anti-sense); si-THSD7A, 5′-GGGCAUUUGUGUUACUCAUTT-3′ (sense), and 5′-AUGAGUAACACAAAUGCCCTT-3′ (anti-sense); miR-210 mimics, 5′–CUGUGCGUGUGACAGCGGCUGA-3′ (sense), and 5′- AGCCGCUGUCACACGCACAGUU-3′(anti-sense); Anti-miR-210, 5′-UCAGCCGCUGUCACACGCACAG-3′ (sense); Anti-NC, 5′-CAGUACUUUUGUGUAGUACAA-3′ (sense).

To generate luciferase reporter plasmids for THSD7A, 3′UTR segments of human THSD7A mRNA (10321–10500nt, Genbank accession no. NM_015204.2) containing the putative miR-210 binding sequence were amplified and cloned into pMIR-REPORT Luciferase plasmid (Ambion, Austin, Texas, USA) at the Mlu1 and Spe1 sites. The construct was named as BD-WT. The mutated pMIR-REPORT plasmid, which carries site mutations in the 3′UTR segments of human THSD7A mRNA being complementary to the seed sequence of miR-210, was generated based on BD-WT plasmids using Quick Change Lightning Site-Directed Mutagenesis Kit according to the manufacture’s instruction (Stratagene, La Jolla, California, USA). The construct was named as BD-MUT. The primers for vectors construction are: THSD7A BD-WT construct, (forward) 5′-GACTAGTGGAGGCAGCTGTCTAAGAATT-3′, and (reverse)5′-CGACGCGTCATCTTCACATGCCATCTGT-3′; THSD7A BD-MUT construct, (forward)5′-ACATTTCCTTTTTAACCGTCAAAATGTCTATGTACAA-3′, and (reverse) 5′-TTGTACATAGACATTTTGACGGTTAAAAAGGAAATGT-3′. All the constructs were confirmed by DNA sequencing.

### Cell culture and treatment

Immortalized human trophoblast cell line, HTR8/SVneo, was a kind gift from Dr. CH Graham at Queen’s University, Canada^57^. The cells were cultured in RPMI1640 medium (Life technologies, CA, USA) supplemented with 10% foetal bovine serum (FBS) and were passaged at a ratio of 1:5 every 3 days.

For transient transfection experiments, the cells were seeded in 6-well plates at 1.5 × 10^5^ cells per well in complete medium. Twenty-four hours following seeding, miR-210 mimics, anti-miR-210 and specific siRNA for THSD7A were transfected into the cells using Lipofectamine 2000 reagent according to the manufacturer’s instruction (Invitrogen, Carlsbad, CA). Cells were incubated under normoxic (20%O_2,_ 5% CO_2_, and 75% N_2_) or hypoxic (2% O_2_, 5% CO_2_, and 93%N_2_) condition for certain time period and subjected to transwell invasion assay or RNA/protein extraction. The culture media were preconditioned to the corresponding O_2_ level, and the oxygen concentrations in the incubators were continuously monitored.

### Transwell Insert Invasion Assay

The invasion assay was conducted in 24-well fitted inserts with membranes (8 μm pore size; Costar, Cambridge, MA, USA) as reported previously^58^. 48 hours after transfection, cells were treated with 10 μg/ml Mitomycin C for another 2 h, and were trypsinized and seeded into transwell insert pre-coated with 200 μg/ml matrigel (BD Biosciences, USA) at 5 × 10^4^ cells per insert. The top chambers contained RPMI 1640 medium supplemented with 1% FBS, and the lower chambers were loaded with RPMI1640 medium plus 10% FBS. The cells were fixed and stained with haematoxylin at 28 hours later. Cells on the upper surface of the membrane were completely removed, and the number of stained cells at the lower surface of the membrane was counted under light microscope. The invasion index was expressed as the percentage of invaded cell number compared with the corresponding control.

### Quantitative real time PCR (qRT-PCR)

Total RNA was extracted from the cells or the placental tissues using TRIzol (Invitrogen) following the manufacturer’s instruction. qRT-PCR was performed using a Roche Light Cycler480 II detection system (Roche, Basel, Switzerland). For the quantification of miRNA cDNA, the reaction was performed according to the instruction of the miRcute miRNA qRT-PCR detection kit (Tiangen, Beijing, China). The reaction for each sample was carried out in duplicates with an initial denaturing at 94 °C for 2 min, followed by 40 cycles of 94 °C for 20 s, 60 °C for 30 s and 72 °C for 30 s. The detection of cDNA was carried out following the instructions of the SYBR® Premix Ex TaqTM kit (Takara, Dalian, China), and the reaction for each sample was carried out in duplicate at 95 °C for 30 s, followed by 40 cycles of 95 °C for 5 s, 60 °C for 31 s. Relative expression level of THSD7A was normalized to the value of glyceraldehyde-3-phosphate dehydrogenase (GAPDH) and miR-210 was normalized to U6. The sequences of primers are: THSD7A, 5′-GTGGAGGGATGGACTACACTG-3′(forward) and 5′-TGCCAATCGCAAACTTTGAAAC-3′(reverse); GAPDH, 5′-AAGGTCATCCCTGAGCTGAAC-3′(forward) and 5′-ACGCCTGCTTCACCACCTTCT-3′(reverse); hsa-miR-210, 5′-CGTGTGAGAGCGGCTGAAA-3′(forward); U6, 5′-CGCAAGGATGACACGCAAATTC-3′ (forward). The relative mRNA/miRNA level was calculated by the 2^−ΔCT^ method^59^, and ΔCT indicated the subtraction of the CT value for GAPDH or U6 from the CT value for the interest mRNA or miRNA.

### Western blot analysis

Whole lysates from HTR8/SVneo cells or the placental tissues were extracted with RIPA buffer containing 150 mM NaCl, 10 mM Tris (pH7.6), 1% NP-40, 0.5% Na deoxylcholate, 0.1% SDS, 1 mM NaF, Na_3_VO_4_ and protease inhibitor cocktail (Sigma Aldrich, MO, USA). Protein concentration was measured using the BCA™ Protein Assay Kit (PIERCE). Lysates were resolved on SDS-PAGE gels and then transferred to nitrocellulose membrane (GE Healthcare, CT, USA). The antibodies used included goat polyclonal antibody against THSD7A (R&D Systems, SANTA, USA) and mouse monoclonal antibody against GAPDH (Ambion, Austin, Texas, USA). The signals were visualized using ECL kit (Thermo Scientific, MA, USA) followed by exposure to X-ray films (Kodak, NY, USA) and analysis by the Quantity One® 1-DAnalysis Software, Version 4.4 (Bio-Rad, CA, USA). The relative density of THSD7A was measured by comparing its densitometry values with that of GAPDH in the same blot.

### Immunohistochemistry

Paraffin sections of 5 μm in thickness were subjected to routine rehydration and antigen retrieval before being incubated with antibody against cytokeratin8 (CK8,1:300; Novus Biologicals, Novus, USA) or THSD7A (1:50; R&D, SANTA, USA). The sections were further incubated with secondary antibody conjugated with horseradish peroxidase (Zhongshan Goldenbridge, Beijing, China) and were visualized with diaminobenzidine (Zhongshan Goldenbridge, Beijing, China) as a substrate.

### Luciferase reporter assay

HTR8/SVneo cells were transfected with 100 ng pMIR-REPORT construct that carries the wild type or the mutant THSD7A 3′UTR (BD-WT or BD-MUT), together with 10 ng pRL-TK control vector (encoding Renilla luciferase) and 40 nM miR-210 mimics or scramble control (NC). 48 hours after transfection, activities of Firefly and Renilla luciferase were measured using Dual-Glo Luciferase Assay System (Promega) and a GloMax 96 microplate luminometer (Promega). The Firefly luciferase activity was normalized to the Renilla luciferase activity.

### Statistical Analysis

Data of transwell insert invasion assay, dual luciferase assay, real-time PCR and Western blotting are shown as the mean ± SEM based on three independent experiments. Statistical analysis was performed using one-way ANOVA or two-sided Student’s t test, and differences were considered significant at *P* < 0.05. All statistical analyses were conducted using GraphPad Prism version 6.01 for Windows (GraphPad Software, San Diego, CA).

## Additional Information

**How to cite this article**: Luo, R. *et al.* Hypoxia-inducible miR-210 contributes to preeclampsia via targeting thrombospondin type I domain containing 7A. *Sci. Rep.* **6**, 19588; doi: 10.1038/srep19588 (2016).

## Supplementary Material

Supplementary Information

## Figures and Tables

**Table 1:** Clinical characteristics of study patients.

Characteristics	Normal pregnancy(n = 22)	Preeclampsia(n = 13)	*P*-value
Maternal age (years)	31.72 ± 4.55	29.33 ± 4.82	0.154
BMI (kg/m2)^a^	22.14 ± 2.51	21.62 ± 3.03	0.770
Systolic blood pressure (mmHg)	116.42 ± 5.83	156.41 ± 12.63*	<0.001
Diastolic blood pressure (mmHg)	75.92 ± 5.04	101.30 ± 7.61*	<0.001
50 g GCT (mmol/L)^b^	7.32 ± 1.54	6.73 ± 1.04	0.226
24 h urine protein (g)	0.00	4.34 ± 0.40*	<0.001
Primiparous percentage (%)	72.73	61.54	NA
Gestational day at delivery (Day)	268.23 ± 7.78	255.62 ± 5.72	0.058
Infant birth weight (g)	3340.31 ± 494.42	2587.04 ± 581.93*	0.003

Data are shown as the mean ± SD, and significant difference between groups was analyzed with one-way ANOVA. *compared with normal pregnancy, *P* < 0.05.

^a^BMI, body mass index, indicating the weight in kilograms divided by the square of the height in meters.

^b^GCT, glucose challenge test.
