# Supplementary material for: Hypoxia-inducible miR-210 contributes to preeclampsia via targeting thrombospondin type I domain containing 7A
Source: Sci Rep. 2016 Jan 22;6:19588. doi: 10.1038/srep19588 (PMC4726282; doi:10.1038/srep19588)
Supplement: Supplementary Information [file srep19588-s1.doc]

**Hypoxia-inducible miR-210 contributes to preeclampsia via targeting thrombospondin type I domain containing 7A**

Rongcan Luo^1,4,6,a^, Yongqing Wang^2,a^, Peng Xu^1,5,a^, Guangming Cao^1,6^, Yangyu Zhao^2^, Xuan Shao^1^, Yu-xia Li^1^, Cheng Chang^4^, Chun Peng^3^, and Yan-ling Wang^1,*^

1. State Key Laboratory of Reproductive Biology, Institute of Zoology, Chinese Academy of Sciences, Beijing 100101, China;

2. Department of Obstetrics and Gynaecology, Peking University Third Hospital, Beijing 100191, China;

3. Department of Biology, York University, Toronto, Ontario, Canada;

4. School of Life Sciences, Lanzhou University, Lanzhou 730000, China;

5. College of Life Sciences, Shanxi University, Taiyuan, Shanxi 030006, China;

6. University of Chinese Academy of Sciences, Beijing 100049, China;

a. These authors have equal contribution.

* All correspondence and reprint requests should be addressed to Dr. Yan-ling Wang at the State Key Laboratory of Reproductive Biology, Institute of Zoology, Chinese Academy of Sciences, 1 Beichen West Road, Chaoyang District, Beijing 100101, China (Tel/Fax: +86-10-64807195, Email: wangyl@ioz.ac.cn).


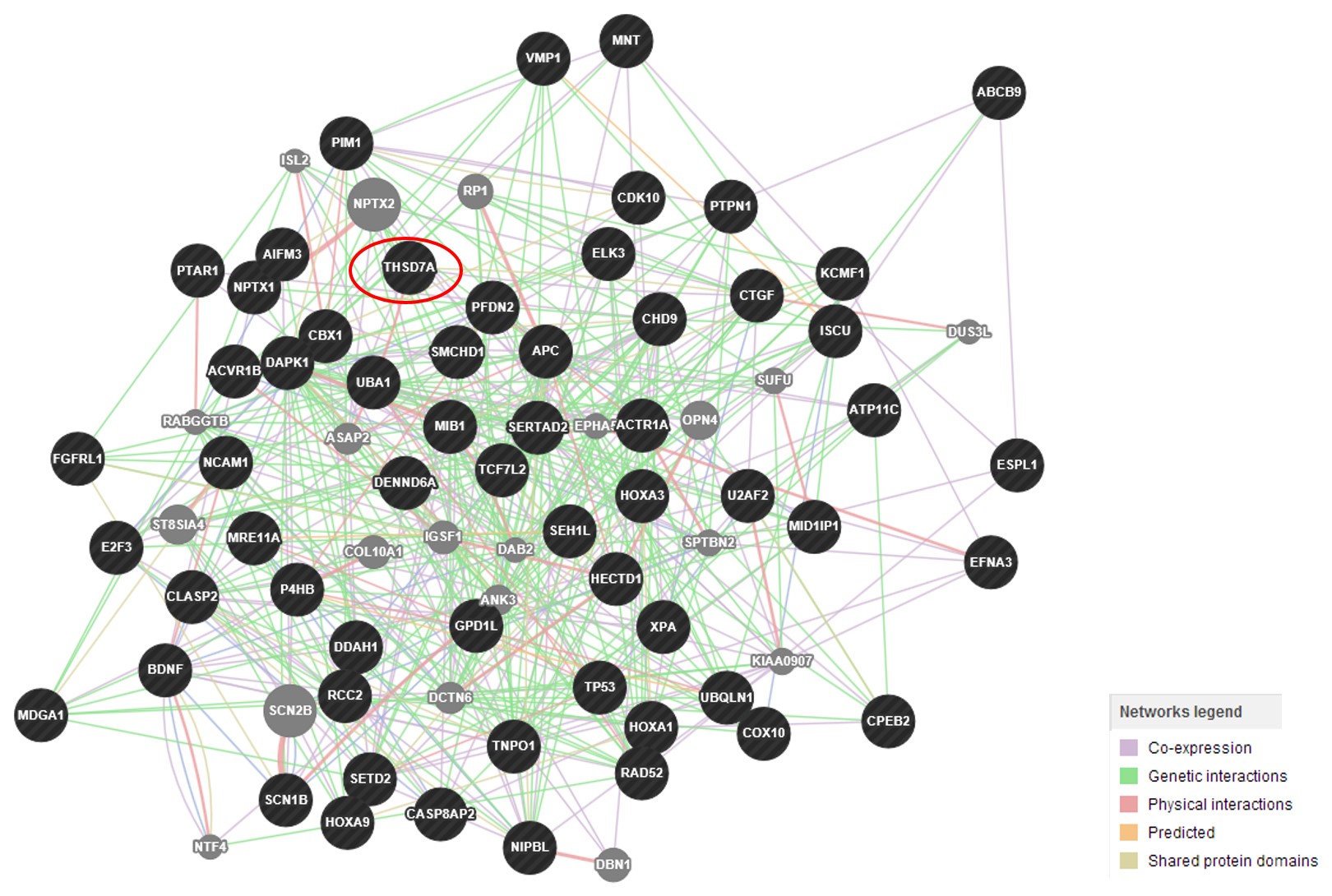


Supplementary figure 1. **Composite network for THSD7A**. MiR-210 target genes and potential target genes were included in the composite network for THSD7A. Association network integration was performed by GeneMANIA (http://beta.genemania.org:8081/genemania/) Argetentary figure 2THSD7A0000000000000000000000000000000000000000000000000000000000000000000000000000000000000000000000000000


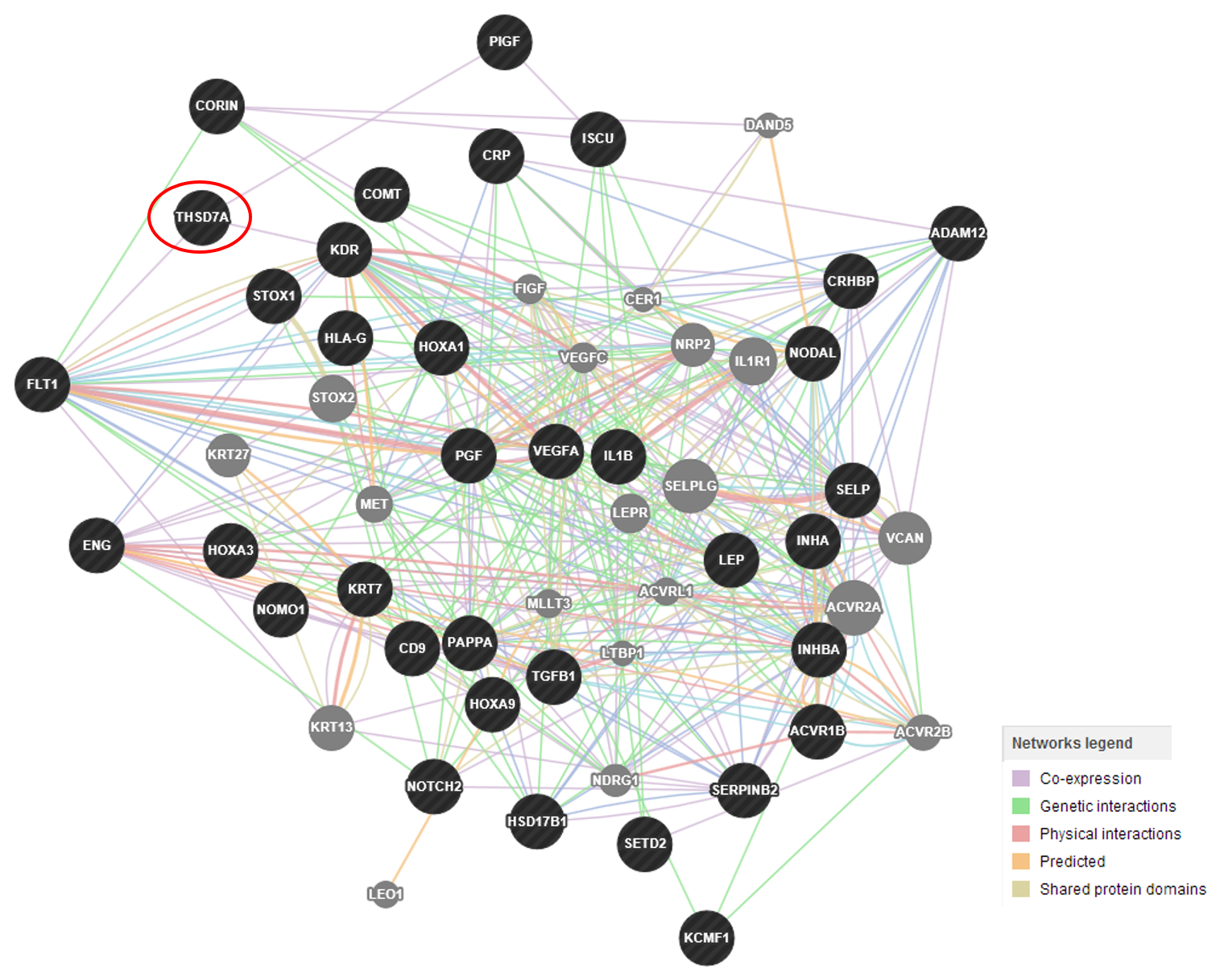


Supplementary figure 2. **Bioinformatic analysis of THSD7A links with other miR-210 targets or the known preeclampsia-associated genes**. Bioinformatic analysis was performed by GeneMANIA (http://beta.genemania.org:8081/genemania/).
